# Age- and sex-dependent associations of metabolic syndrome with colorectal cancer in the UK Biobank

**DOI:** 10.1038/s41416-026-03564-1

**Published:** 2026-07-30

**Authors:** Zilin Luo, Fatemeh Safizadeh, Marko Mandic, Michael Hoffmeister, Hermann Brenner

**Affiliations:** 1 Clinical Epidemiology of Early Cancer Detection, German Cancer Research Center (DKFZ), Heidelberg, Germany; 2 Office of Cancer Screening, National Cancer Center/National Clinical Research Center for Cancer/Cancer Hospital, Chinese Academy of Medical Sciences and Peking Union Medical College, Beijing, China; 3 Medical Faculty Heidelberg, Heidelberg University, Heidelberg, Germany; 4 Cancer Prevention Graduate School, German Cancer Research Center (DKFZ), Heidelberg, Germany

**Keywords:** Cancer epidemiology, Cancer prevention, Metabolic syndrome

## Abstract

**Background:**

The concurrent rise in metabolic syndrome (MetS) and the changing age profile of colorectal cancer (CRC) necessitates clarifying the heterogeneity in their association across demographic groups. This study aimed to define the precise age- and sex-specific associations between MetS and CRC risk, addressing a key gap to inform targeted prevention paradigms.

**Methods:**

In this cohort study, we analysed data from UK Biobank participants aged 40–69 years (recruited 2006–2010). Participants were stratified by age (<60 or ≥60 years) and sex into four groups. Multivariable Cox proportional hazards models were used to assess the associations of MetS, the number of metabolic abnormalities, and each component with CRC incidence.

**Results:**

Among 379,173 participants, 4971 developed CRC during a median follow-up of 11.8 years. MetS was most strongly associated with CRC in younger males (hazard ratio [HR] 1.31, 95% CI 1.15–1.50), significantly in older adults of both males (HR 1.18, 95% CI 1.07–1.30) and females (HR 1.18, 95% CI 1.05–1.33), but not in younger females (HR 1.05, 95% CI 0.89–1.25). Older males but not females showed increasing risks when having 3, 4, and 5 abnormalities compared to none, respectively. Beyond younger females, elevated waist circumference was the sole component consistently linked to higher risk.

**Conclusions:**

The association between MetS and CRC is strongly dependent on age and sex in White populations, with the highest risk concentrated in young and middle-aged males. These findings directly support risk-stratified prevention and provide crucial clues for investigating the underlying sex- and age-specific biological mechanisms.

## Introduction

Colorectal cancer (CRC) ranked as the third most commonly diagnosed cancer and the second leading cause of cancer-related mortality globally in 2022 [1]. A particularly alarming trend is the rapidly increasing incidence of CRC in younger populations [2]. Despite extensive research, the factors driving this changing age profile of CRC remain incompletely understood.

Metabolic syndrome (MetS), a cluster of interrelated metabolic abnormalities including central obesity, hypertriglyceridemia, hypertension, hyperglycaemia, and dyslipidemia [3], is a well-established risk factor for CRC [4]. Emerging evidence has linked MetS to CRC risk specifically in younger populations. A nested case-control study from the US reported a 25% higher risk of early-onset CRC (diagnosed before age 50) among individuals with MetS, with females showing a more pronounced 32% increased risk compared to males’ 19% elevated risk [5]. Similarly, a large South Korean prospective cohort study with a median 9-year follow-up, involving over 5.7 million adults aged 20–49, demonstrated that MetS was associated with significantly elevated CRC risk, showing hazard ratios ranging from 1.14 to 1.29 depending on tumour location [6].

While existing studies have examined MetS-related CRC risk through either sex or age stratification, few investigations have employed joint stratification by both factors. Consequently, the potential interaction between sex-specific biological mechanisms and age-dependent susceptibility in MetS-associated carcinogenesis remains poorly understood. This gap is especially important given the biological, hormonal, and behavioural differences across different ages and between males and females. Understanding how these factors interact is vital for improving individualised risk prediction and tailoring CRC screening strategies.

Therefore, this study aims to evaluate the association between MetS and CRC risk across sex-specific and age-specific subgroups, leveraging the rich prospective cohort data from the UK Biobank.

## Methods

### Study design and population

This study was based on a large-scale, population-based prospective cohort derived from the UK Biobank, which recruited over 500,000 participants aged 40–69 years between 2006 and 2010. The details have been previously documented [7]. In brief, participants were enroled from 22 assessment centres across England, Scotland, and Wales and underwent comprehensive baseline assessments, including questionnaires, physical measurements, and biological sample collection. Longitudinal follow-up of participants was conducted through linkage with electronic health records, enabling continuous tracking of various health outcomes including cancer incidence. Ethical approval for the UK Biobank study was obtained from the North West Multi-Centre Research Ethics Committee (11/NW/0382). All participants provided written informed consent prior to enrolment. Individuals with a prior cancer diagnosis (except for non-melanoma skin cancer) or missing data on MetS components were excluded from the current analysis. This study adhered to the Strengthening the Reporting of Observational Studies in Epidemiology (STROBE) reporting guideline [8].

### Assessment of MetS

MetS was defined according to established criteria [3], incorporating five metabolic abnormalities: elevated waist circumference (≥102 cm in males and ≥88 cm in females for European populations), hypertriglyceridemia (≥1.7 mmol/L), hyperglycaemia (glycated haemoglobin (HbA1c) ≥ 42 mmol/mol, or antidiabetic medication use), elevated blood pressure (systolic ≥130 mmHg, diastolic ≥85 mmHg, or antihypertensive medication use), and low HDL-C (<1.03 mmol/L in males, <1.29 mmol/L in females, or lipid-modifying medications) (Table S1). For hyperglycaemia, because the UK Biobank study did not require fasting before blood glucose measurement, we used the more stable indicator of HbA1c, with a cutoff of ≥42 mmol/mol to define impaired glucose regulation [9, 10].

Lipid-modifying medications can influence multiple aspects of the lipid profile, including HDL-C and triglycerides [11]. To mitigate potential redundancy in classification, these medications were categorised under the reduced HDL-C group. The use of these medications was determined through Anatomical Therapeutic Chemical (ATC) codes, referencing an extensive review of prior studies that defined MetS using ATC classifications [10, 12–14]. A diagnosis of MetS was assigned when at least three of these criteria were met.

### Outcomes

Incident CRC cases were identified through linkage to national cancer registries and hospitals, using International Classification of Diseases, 10th Revision (ICD-10) codes C18–C20. Follow-up data for cancer incidence were accessible through national cancer registries until July 31, 2019, for England, December 31, 2016, for Wales, and October 31, 2015, for Scotland. CRC cases diagnosed beyond these respective registry censoring dates were captured through hospital episode statistics, which remained available until September 30, 2021, for England and July 31, 2021, for Scotland. In Wales, complete diagnostic records were only obtainable up to March 31, 2016, preceding the registry data cutoff date.

### Covariates

During baseline evaluations, comprehensive covariate data were collected through standardised protocols. Demographic variables included age (continuous variable), self-reported ethnicity (categorical: White, Other), and socioeconomic status measured by the Townsend deprivation index (continuous). Educational attainment was classified as an ordinal categorical variable (higher academic/professional, lower academic/vocational, no formal qualifications). Lifestyle factors comprised smoking status (categorical: never, former, current), alcohol consumption frequency (categorical: never, special occasions only, 1–3 times/month, 1–2 times/week, 3–4 times/week, daily or almost daily), and physical activity levels (categorical: low, moderate, high based on international physical activity questionnaire (IPAQ) [15]. Dietary variables included daily fruit intake (continuous, pieces/day) and vegetable consumption (continuous, tablespoons/day), along with red/processed meat intake (categorical: never, less than once a week, once a week, ≥2 times a week). Medical covariates were all categorical: CRC screening history (binary), family history of CRC in first-degree relatives (binary), and regular use of non-steroidal anti-inflammatory drugs (NSAIDs) or aspirin (binary).

### Statistical analysis

In the UK Biobank, incident early-onset CRC cases were too few for meaningful analysis [16]. As the aim of the present study was not to address early-onset colorectal cancer specifically, an age cutoff of 60 years was selected to ensure adequate power and precision. This cutoff balanced the numbers of participants and CRC events across comparison groups, a prerequisite for adequate power to assess interaction.

Accordingly, four subgroups were defined based on sex and the 60-year cutoff: younger males (age <60 years), older males (age ≥60 years), younger females (age <60 years), and older females (age ≥60 years), and conducted stratified analyses accordingly. Baseline characteristics were summarised using means (standard deviations) for continuous variables and proportions for categorical variables. Group comparisons were conducted using Pearson’s Chi-squared test or Kruskal-Wallis rank sum test as appropriate.

To describe the distribution of MetS biomarkers, we assessed the sex- and age-stratified prevalence of MetS and its individual components. Age was grouped in 5-year intervals, and the prevalence of MetS and its components was examined across these age categories separately for males and females to evaluate sex-stratified age trends. Furthermore, we explored the variations in the number of metabolic abnormalities by sex-by-age stratification to depict how the accumulation of metabolic abnormalities differed across demographic subgroups.

Multivariable Cox proportional hazards models were employed to estimate hazard ratios (HRs) and 95% confidence intervals (CIs) for the association between MetS and CRC risk. Proportional hazards assumptions were verified using Schoenfeld residuals. All models adjusted for the full range of measured covariates, with additional adjustment for sex in the overall population analyses. To quantify the contribution of MetS to CRC risk, we calculated population attributable fractions (PAFs) with 95% CIs using Eide and Gefeller’s method [17], maintaining covariate consistency with the HR calculation. The adjusted PAFs were derived through logistic regression by systematically permuting the entry order of risk factors, with final estimates representing the mean values from all possible combinations computed using the ‘averisk’ R package, which generated CIs via Monte Carlo simulation [18].

To examine potential heterogeneity by tumour subsite, stratified analyses were performed for proximal colon, distal colon, and rectum, separately. Similarly, stratified analyses by family history of CRC in first-degree relatives were conducted. Sensitivity analyses were performed by excluding the first 1, 2, 3, and 4 years of follow-up, respectively, to minimise reverse causality [19].

We also examined the association between the number of metabolic abnormalities (ranging from 1 to 5, with 0 as the reference) and CRC risk, and tested for linear trends across categories to evaluate a potential dose–response relationship. To explore the individual contributions of each component, Cox models were constructed by entering each MetS component separately. Interaction terms between MetS and sex, age group (<60 vs. ≥60 years), and their combinations were tested in fully adjusted models to assess potential modification associations. Post-hoc power analyses were conducted to assess whether the sample size was sufficient to detect these interactions.

Moreover, we evaluated the potential nonlinear associations between six continuous variables (derived from five MetS components: waist circumference, triglycerides, HbA1c, systolic blood pressure, diastolic blood pressure, and HDL-C) and CRC risk across four stratified subgroups. Values below the 1st percentile or above the 99th percentile were treated as outliers and excluded from the analysis [10]. Restricted cubic spline (RCS) models were used to explore nonlinear patterns, using the median of each variable as the reference point and adjusting for all relevant covariates.

All statistical procedures were conducted in R software (version 4.4.1). Missing covariate data were imputed using the multiple imputation technique via the mice package. We generated five imputed datasets, whose results were subsequently pooled. Statistical significance was defined as two-sided *P* < 0.05.

## Results

### Baseline characteristics

**Fig. 1:**
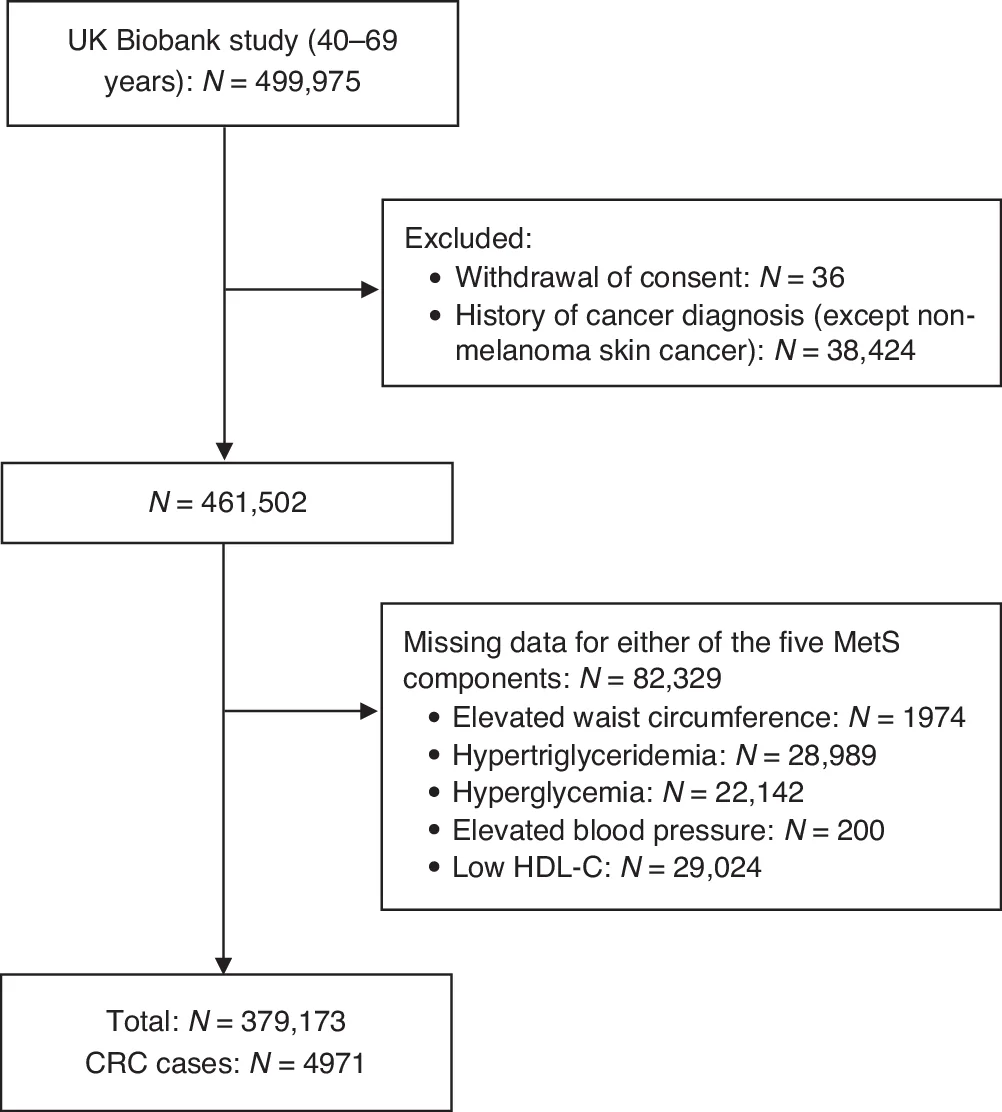
Study flow chart. Abbreviations: MetS metabolic syndrome, HDL-C high-density lipoprotein cholesterol, CRC colorectal cancer.

The selection flowchart for the study population is presented in Fig. 1. Among the 379,173 participants included in the analysis, 4971 were diagnosed with CRC over a median follow-up of 11.8 years (interquartile range: 11.1–12.5). Table S2 shows that the participants were divided into four subgroups by sex and age: younger males (*n* = 101,415, 26.7%), older males (*n* = 78,677, 20.7%), younger females (*n* = 117,078, 30.9%), and older females (*n* = 82,003, 21.6%). The highest incidence was observed in older males (1907 cases, incidence rate: 208.2/100,000 person-years). Younger females had the lowest CRC incidence (815 cases, incidence rate: 59.1/100,000 person-years). Notably, Table 1 demonstrates that MetS exposure differed significantly between CRC and non-CRC groups in all subgroups except for younger females (all *p* < 0.001).

**Table 1:** Metabolic syndrome-related characteristics in participants with and without colorectal cancer stratified by sex and age.

	Male, age <60		Male, age ≥60		Female, age <60		Female, age ≥60	
	Non-CRC	CRC	Non-CRC	CRC	Non-CRC	CRC	Non-CRC	CRC
	(*n* = 100,453)	(*n* = 962)	(*n* = 76,770)	(*n* = 1907)	(*n* = 116,263)	(*n* = 815)	(*n* = 80,716)	(*n* = 1287)
MetS	30,293 (30.2%)	367* (38.1%)	32,339 (42.1%)	883* (46.3%)	24,412 (21.0%)	192 (23.6%)	28,160 (34.9%)	506* (39.3%)
Waist circumference, cm	96.2 ± 11.5	99.0 ± 12.3*	98.0 ± 11.1	99.8 ± 11.2*	83.7 ± 12.8	84.8 ± 12.1*	86.0 ± 12.1	86.9 ± 12.6*
HbA1c, mmol/mol	35.7 ± 7.4	37.0 ± 9.3*	37.6 ± 7.8	37.7 ± 7.7	34.9 ± 5.8	35.4 ± 7.0*	37.1 ± 6.1	37.4 ± 5.9*
Blood pressure, mmHg								
Systolic	137.5 ± 16.0	140.8 ± 16.7*	145.2 ± 18.2	146.6 ± 18.2*	130.2 ± 17.6	133.4 ± 18.7*	142.3 ± 19.1	142.6 ± 19.7
Diastolic	84.4 ± 10.1	86.2 ± 10.2*	83.7 ± 9.9	83.8 ± 9.9	80.4 ± 10.1	81.4 ± 10.1*	81.1 ± 9.8	81.1 ± 9.7
HDL-C, mmol/L	1.3 ± 0.3	1.3 ± 0.3	1.3 ± 0.3	1.3 ± 0.3	1.6 ± 0.4	1.6 ± 0.4	1.6 ± 0.4	1.6 ± 0.4
Triglycerides, mmol/L	2.0 ± 1.2	2.1 ± 1.4	1.9 ± 1.1	2.0 ± 1.0*	1.4 ± 0.8	1.6 ± 0.9*	1.7 ± 0.9	1.8 ± 0.9*
Glucose-lowering medication	3943 (3.9%)	72* (7.5%)	5360 (7.0%)	157* (8.2%)	2368 (2.0%)	21 (2.6%)	2907 (3.6%)	50 (3.9%)
Antihypertensives	16,140 (16.1%)	205* (21.3%)	30,362 (39.5%)	803* (42.1%)	14,217 (12.2%)	129* (15.8%)	24,549 (30.4%)	407 (31.6%)
Medications for low HDL-C	13,521 (13.5%)	185* (19.2%)	27,765 (36.2%)	735* (38.5%)	7809 (6.7%)	79* (9.7%)	17,911 (22.2%)	291 (22.6%)

Data are presented as no. (%) or mean ± (SD), unless otherwise indicated. Comparisons between CRC and non-CRC groups within each subgroup were performed using Pearson’s chi-squared test for categorical variables and Wilcoxon rank sum test for continuous variables. *p < 0.05.

*CRC* colorectal cancer, *MetS* metabolic syndrome, *HbA1c* glycated haemoglobin, *HDL-C* high-density lipoprotein cholesterol.

Figure S1 illustrates that MetS prevalence increased with age in both sexes, rising from 23.7% (40–44 years) to 44.6% (65–69 years) in males and from 14.2% to 38.5% in females. Notably, the sex disparity in MetS prevalence narrowed with advancing age: at 40–44 years, males had a 67% higher prevalence than females, whereas by 65–69 years, the difference reduced to only 16%. Figure S2 reveals that older males showed the highest prevalence of 3-5 metabolic abnormalities (24.5%, 13.4% and 4.3%, respectively). Figure S3 indicates that all MetS components except hypertriglyceridemia demonstrated increasing prevalence with age in both sexes, with the steepest rises observed for hyperglycaemia (from 4.0% to 15.5% in males, 2.1% to 11.6% in females). Most MetS components showed higher prevalence in males, except for elevated waist circumference which was consistently more prevalent in females across all age groups.

### Metabolic syndrome and colorectal cancer

**Fig. 2:**
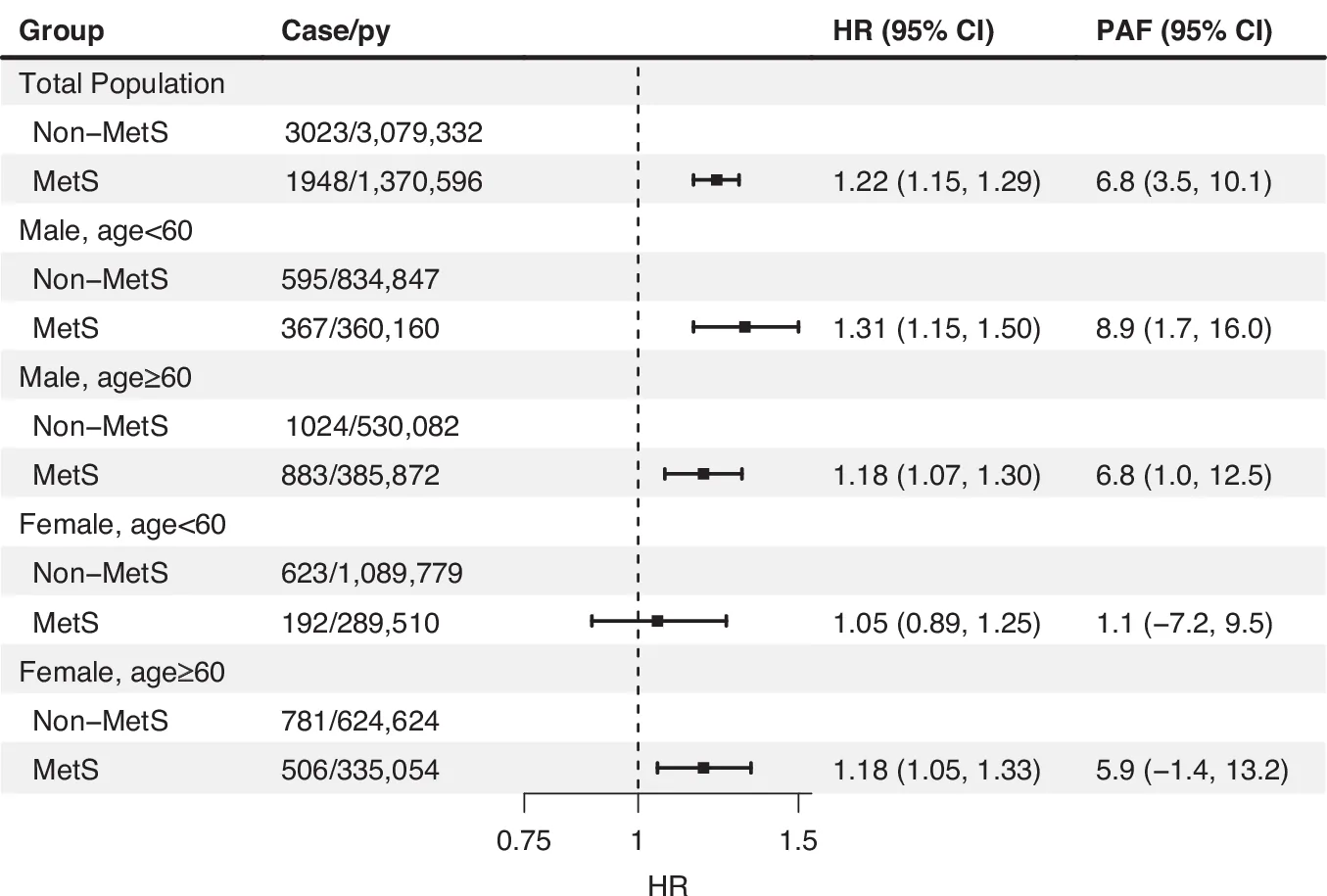
Forest plot of overall and sex- and age-specific associations between metabolic syndrome and colorectal cancer risk. Models were adjusted for age, sex (only in the analysis of total population), ethnicity, socio-economic deprivation, education, smoking status, alcohol consumption, physical activity, fruit, vegetable, red meat and processed meat intake, history of bowel cancer screening, family history of CRC, and regular use of NSAIDs. Abbreviations: py person-years, HR hazard ratio, CI confidence interval, PAF population attributable fractions, MetS metabolic syndrome.

Figure 2 demonstrates that MetS showed a significant association with increased CRC risk in the overall population (HR 1.22, 95% CI 1.15–1.29), with a PAF of 6.8% (95% CI, 3.6–10.0). Stratified analyses revealed important variations across sex and age subgroups. The strongest association was observed in younger males (HR 1.31, 95% CI 1.15–1.50), accounting for 8.9% (95% CI, 1.1–16.7) of attributable risk in this subpopulation. In contrast, younger females showed no significant association (HR 1.05, 95% CI 0.89–1.25; PAF 1.1%, 95% CI [−7.4]–9.7). Among older adults, significant but more modest associations were found in both older males (HR 1.18, 95% CI 1.07–1.30; PAF 6.8%, 95% CI 1.0–12.5) and older females (HR 1.18, 95% CI 1.05–1.33; PAF 5.9%, 95% CI [−0.5]–12.3).

Further stratification by tumour subsite (Fig. S4) showed that the overall association remained significant across proximal colon, distal colon, and rectal cancers, consistent with the main analysis. The strongest effect persisted in younger males for proximal and distal colon cancers. However, for rectal cancer, the association was attenuated across all subgroups, with no statistically significant findings observed in any of the four sex-age subgroups.

Stratification by family history of CRC (Fig. S5) revealed that, in the overall population, significant associations were present regardless of family history. Notably, the sex- and age-specific pattern differed substantially between the two strata. Among individuals without a family history, the results mirrored the main analysis, with the strongest association confined to younger males. In contrast, among those with a family history, the association in younger males was no longer significant, whereas significant associations emerged in younger females and older males.

Figure S6 presents the results of sensitivity analyses excluding the first 1–4 years of follow-up. It confirmed the robustness of these findings overall, though the association became non-significant in older females when excluding the first 3 years (HR 1.13, 95% CI 0.99–1.29) or the first 4 years (HR 1.11, 95% CI 0.97–1.27). Table S3 shows that all tested interaction terms (MetS × sex, MetS × age group, and MetS × sex × age group) were non-significant (all *p* > 0.05), though these analyses were limited by low statistical power (post-hoc power <80%).

### Metabolic syndrome markers and colorectal cancer

**Fig. 3:**
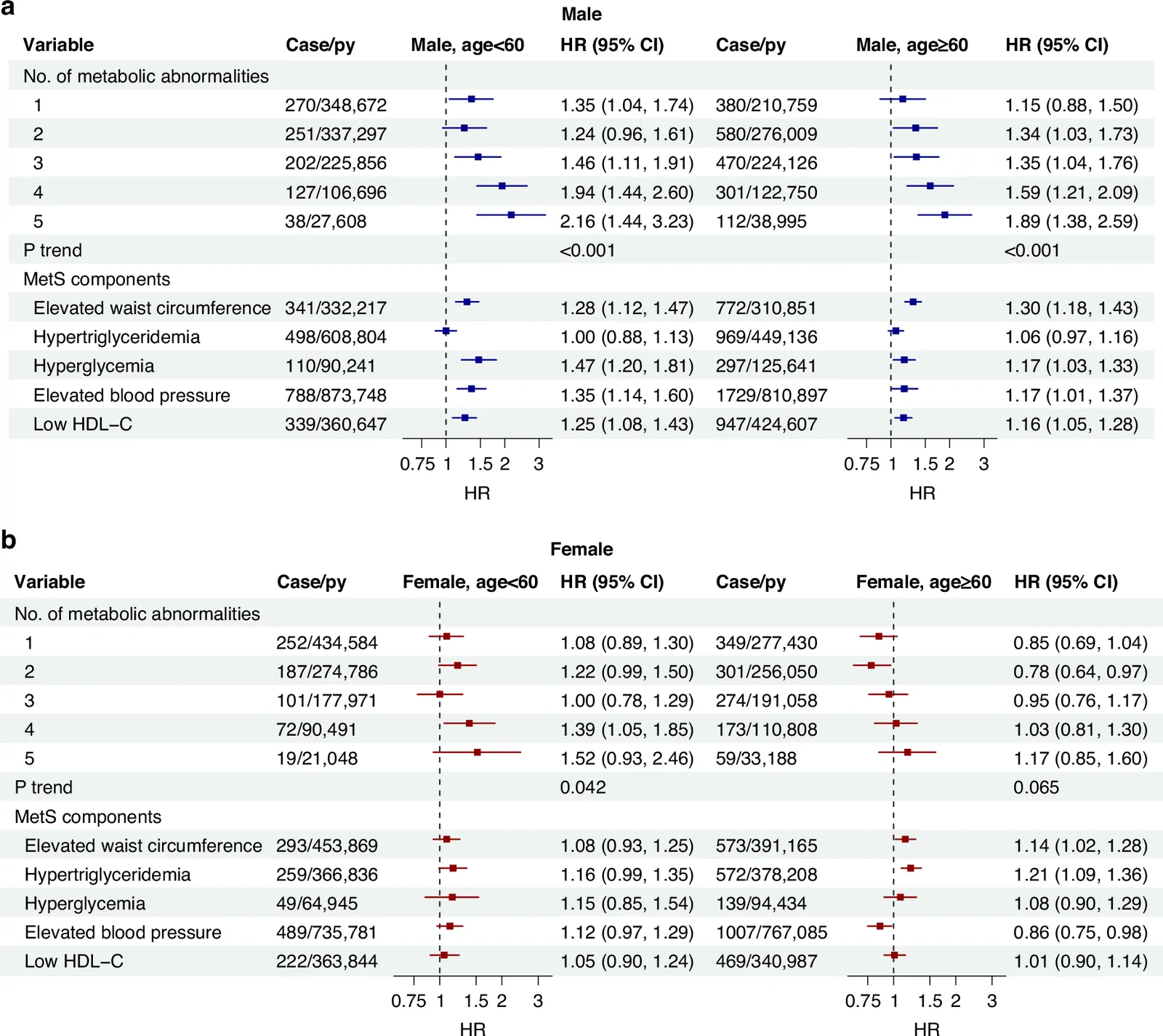
Forest plot of sex- and age-specific associations between metabolic syndrome markers and colorectal cancer risk. **a** Male; (**b**) Female. Models were adjusted for age, sex (only in the analysis of total population), ethnicity, socio-economic deprivation, education, smoking status, alcohol consumption, physical activity, fruit, vegetable, red meat and processed meat intake, history of bowel cancer screening, family history of CRC, and regular use of NSAIDs. Abbreviations: py person-years, HR hazard ratio, CI confidence interval, MetS metabolic syndrome, HDL-C high-density lipoprotein cholesterol.

Figure 3 displays distinct sex- and age-specific patterns in the association between metabolic abnormalities and CRC risk. In males across both age groups, a consistent dose-response relationship was observed, with increasing numbers of metabolic abnormalities significantly elevating CRC risk (younger males: HR for 5 abnormalities = 2.16, 95% CI 1.44–3.23, *P* for trend <0.001; older males: HR = 1.89, 1.38–2.59, *P* for trend <0.001). This trend was less pronounced in females, particularly those who are older (*P* for trend = 0.065).

In the individual component analysis, Fig. 3 also reveals that none of the five components demonstrated a statistically significant association with CRC risk in younger females. Elevated waist circumference significantly increased the risk in all other subgroups. Furthermore, hyperglycaemia, hypertension and low HDL-C consistently predicted risk in males (HR range: 1.25–1.47 in younger males and 1.16–1.17 in older males).

Figures S7–S10 show significant nonlinear associations with CRC risk for waist circumference, triglycerides, and HDL-C in specific subgroups (*P* for nonlinearity <0.05). In younger males, CRC risk remained stable when triglycerides were below 1.7 mmol/L, but increased sharply above this level (*P* for nonlinearity = 0.005). In both younger and older males, higher HDL-C was associated with lower CRC risk up to 1.2 mmol/mol, after which the risk plateaued (*P* for overall <0.001 and 0.011, respectively; *P* for nonlinearity = 0.003 and 0.031, respectively).

## Discussion

To our knowledge, this is the first study that reveals notable variations in the association between MetS and CRC risk across sex-by-age strata, with the most pronounced association observed in younger males and no significant association detected in their female counterparts. These findings collectively suggest that the rising incidence of MetS may disproportionately contribute to an elevated CRC incidence, particularly among younger males, highlighting the need for targeted preventive strategies in this high-risk subgroup.

Previous studies have reported conflicting findings regarding sex differences in the MetS-CRC association. A meta-analysis of 23,745,458 participants from 11 prospective cohorts (not including UK Biobank) found comparable risk elevations in both sexes (males: RR 1.23, 95% CI 1.16–1.30; females: RR 1.21, 1.09–1.35) [20], whereas UK Biobank data (*n* = 366,016) showed significant risk only in males (HR 1.27, 1.14–1.42 vs. female HR 1.01, 0.88–1.17) [21]. Regarding age-specific associations, large US and Korean cohorts confirmed that MetS is associated with both early-onset and later-onset CRC to a comparable extent [5, 6]. Interestingly, our sex- and age-stratified results revealed more complex patterns: while MetS conferred a 31% higher CRC risk in younger males, no significant association was observed in their female counterparts. Conversely, both sexes exhibited similar 18% risk elevations in the older age groups. These findings demonstrate substantial heterogeneity in associations that would be obscured without joint sex-age stratification.

Rather than a binary classification, metabolic dysfunction exists on a continuous spectrum of severity. Assessing the number of abnormal components revealed a dose-response relationship in younger males, where those with all five metabolic abnormalities had a 116% higher CRC risk than those with none. This highlights the importance of evaluating overall metabolic health beyond mere MetS status, especially in young and middle-aged males. Despite a similar overall MetS-CRC association in older males and females, only males had a significantly increased risk with 3, 4, or 5 metabolic abnormalities compared to none. This discrepancy may stem from a lower prevalence of multiple abnormalities in older females, reducing statistical power, and from potential compositional differences. Overall, our results emphasise considering the gradient of metabolic dysfunction and its interactions with age and sex.

MetS comprises a heterogeneous cluster of metabolic abnormalities that may contribute differently to CRC risk across sex and age groups [3]. Although meta-analyses have reported consistent associations between obesity and CRC risk in both sexes [4], our study found notable age-specific differences. Central obesity raised CRC risk by about 30% in both younger and older males, but only in older females (14% increase), with no significant association in younger females. These findings align with prior reports of sex-specific risks: MetS increased rectal cancer risk more in males (28%) than females (16%) [20], and waist circumference seemed to significantly predict risk only in males [22]. Early-onset CRC, often rectal or distal [23], is projected to comprise 11% of colon and 23% of rectal cancers by 2030 [24]. Moreover, key MetS components driving CRC risk differed: hyperglycaemia, hypertension, and low HDL-C were primary in older males, while hypertriglyceridemia was more relevant in older females. No individual components significantly affected younger females, aligning with the overall null association in this group. These insights emphasise the need for sex- and age-specific risk assessment and tailored prevention strategies.

While growing evidence links MetS to CRC through mechanisms like IGF-1 signalling, chronic inflammation, and adipokine dysregulation [25, 26], these pathways remain incompletely understood. Interestingly, age and sex may modify this relationship. Our results suggest that MetS may significantly increase CRC risk in males, but not females at younger ages. This may be attributed to the protective role of oestrogen before menopause, which occurs at an average age of 51 years in the UK [27], combined with the long preclinical duration of CRC development. Oestrogen exhibits anti-inflammatory and cancer-protective properties [28, 29], and several cohort studies show that oestrogen or combined hormone replacement therapy lowers CRC risk by 21–43% in postmenopausal females [30–32]. Additionally, fat distribution differences between sexes (visceral in males versus subcutaneous in females) may explain the lower metabolic and inflammatory risk in premenopausal females [33].

Among older individuals, the sex-specific CRC risk associated with MetS seems to diminish. This may reflect postmenopausal oestrogen loss in females, removing its protective effect [30–32], and elevating their MetS-CRC risk to resemble that in males. In older males, however, the weakened association may partly stem from competing mortality risks like cardiovascular disease, which can obscure cancer-specific outcomes in epidemiology. Collectively, these insights highlight the necessity for personalised preventive strategies that consider sex- and age-related biological factors in CRC risk.

Key strengths of this study include the large-scale, well-powered UK Biobank cohort enabling stratified analysis by both sex and age, and comprehensive data permitting rigorous confounder adjustment. Limitations are, first, the unaccounted duration of MetS and temporal metabolic changes, which is challenging given its insidious onset; second, the use of non-fasting blood samples, although we used stable HbA1c for glucose metabolism; and third, potential limited generalisability due to the predominantly (>90%) White cohort composition. Our results align with European studies but contrast with stronger American and null Australian associations [20], highlighting that multi-ethnic relevance should be interpreted with caution.

In summary, this large cohort study underscores critical age and sex disparities in the MetS-CRC risk association in White populations, highlighting younger males as a key subgroup that should be prioritised in targeted public health action.

## Supplementary information

Supplementary Tables

Supplementary Figures

## Data Availability

Data were reused with the permission of the UK Biobank. The UK Biobank is an open-access resource and bona fide researchers can apply to use the UK Biobank dataset by registering and applying at https://www.ukbiobank.ac.uk/enableyourresearch/apply-for-access. The data and analysis codes used for this study are going to be available on the UK Biobank website for registered researchers at the UK Biobank and an application fee.

## References

[1] Bray F, Laversanne M, Sung H, Ferlay J, Siegel RL, Soerjomataram I, et al. Global cancer statistics 2022: GLOBOCAN estimates of incidence and mortality worldwide for 36 cancers in 185 countries. CA Cancer J Clin. 2024;74:229–63. 10.3322/caac.21834 38572751

[2] Akimoto N, Ugai T, Zhong R, Hamada T, Fujiyoshi K, Giannakis M, et al. Rising incidence of early-onset colorectal cancer - a call to action. Nat Rev Clin Oncol. 2021;18:230–43. 10.1038/s41571-020-00445-1 PMC7994182 33219329

[3] Alberti KG, Eckel RH, Grundy SM, Zimmet PZ, Cleeman JI, Donato KA, et al. Harmonizing the metabolic syndrome: a joint interim statement of the International Diabetes Federation Task Force on Epidemiology and Prevention; National Heart, Lung, and Blood Institute; American Heart Association; World Heart Federation; International Atherosclerosis Society; and International Association for the Study of Obesity. Circulation. 2009;120:1640–5. 10.1161/CIRCULATIONAHA.109.192644 19805654

[4] Han F, Wu G, Zhang S, Zhang J, Zhao Y, Xu J. The association of metabolic syndrome and its components with the incidence and survival of colorectal cancer: a systematic review and meta-analysis. Int J Biol Sci. 2021;17:487–97. 10.7150/ijbs.52452 PMC7893592 33613107

[5] Chen H, Zheng X, Zong X, Li Z, Li N, Hur J, et al. Metabolic syndrome, metabolic comorbid conditions and risk of early-onset colorectal cancer. Gut. 2021;70:1147–54. 10.1136/gutjnl-2020-321661 PMC8032822 33037055

[6] Jin EH, Han K, Lee DH, Shin CM, Lim JH, Choi YJ, et al. Association between metabolic syndrome and the risk of colorectal cancer diagnosed before age 50 years according to tumor location. Gastroenterology. 2022;163:637–48.e2. 10.1053/j.gastro.2022.05.032 35643169

[7] Sudlow C, Gallacher J, Allen N, Beral V, Burton P, Danesh J, et al. UK biobank: an open access resource for identifying the causes of a wide range of complex diseases of middle and old age. PLoS Med. 2015;12:e1001779. 10.1371/journal.pmed.1001779 PMC4380465 25826379

[8] Von Elm E, Altman DG, Egger M, Pocock SJ, Gøtzsche PC, Vandenbroucke JP. Strengthening the Reporting of Observational Studies in Epidemiology (STROBE) statement: guidelines for reporting observational studies. Bmj. 2007;335:806–8. 10.1136/bmj.39335.541782.AD PMC2034723 17947786

[9] John WG. Use of HbA1c in the diagnosis of diabetes mellitus in the UK. The implementation of World Health Organization guidance 2011. Diabet Med. 2012;29:1350–7. 10.1111/j.1464-5491.2012.03762.x 22957983

[10] Li M, Cao SM, Dimou N, Wu L, Li JB, Yang J. Association of metabolic syndrome with risk of lung cancer: a population-based prospective cohort study. Chest. 2024;165:213–23. 10.1016/j.chest.2023.08.003 PMC10790176 37572975

[11] Ascaso J, Gonzalez Santos P, Hernandez Mijares A, Mangas Rojas A, Masana L, Millan J, et al. Management of dyslipidemia in the metabolic syndrome: recommendations of the Spanish HDL-Forum. Am J Cardiovasc Drugs. 2007;7:39–58. 10.2165/00129784-200707010-00004 17355165

[12] Yuan F, Wen W, Jia G, Long J, Shu XO, Zheng W. Serum lipid profiles and cholesterol-lowering medication use in relation to subsequent risk of colorectal cancer in the UK biobank cohort. Cancer Epidemiol Biomarkers Prev. 2023;32:524–30. 10.1158/1055-9965.EPI-22-1170 36780218

[13] Mendonça FM, de Sousa FR, Barbosa AL, Martins SC, Araújo RL, Soares R, et al. Metabolic syndrome and risk of cancer: which link? Metabolism. 2015;64:182–9. 10.1016/j.metabol.2014.10.008 25456095

[14] Chandler PD, Song Y, Lin J, Zhang S, Sesso HD, Mora S, et al. Lipid biomarkers and long-term risk of cancer in the Women’s Health Study. Am J Clin Nutr. 2016;103:1397–407. 10.3945/ajcn.115.124321 PMC4880994 27099252

[15] Craig CL, Marshall AL, Sjöström M, Bauman AE, Booth ML, Ainsworth BE, et al. International physical activity questionnaire: 12-country reliability and validity. Med Sci Sports Exerc. 2003;35:1381–95. 10.1249/01.MSS.0000078924.61453.FB 12900694

[16] Gausman V, Liang PS, O’Connell K, Kantor ED, Du M. Evaluation of early-life factors and early-onset colorectal cancer among men and women in the UK Biobank. Gastroenterology. 2022;162:981–3.e3. 10.1053/j.gastro.2021.11.023 PMC8881299 34843734

[17] Eide GE, Gefeller O. Sequential and average attributable fractions as aids in the selection of preventive strategies. J Clin Epidemiol. 1995;48:645–55. 10.1016/0895-4356(94)00161-i 7730921

[18] Ferguson J, Alvarez-Iglesias A, Newell J, Hinde J, O’Donnell M. Estimating average attributable fractions with confidence intervals for cohort and case-control studies. Stat Methods Med Res. 2018;27:1141–52. 10.1177/0962280216655374 27342575

[19] Safizadeh F, Mandic M, Pulte D, Niedermaier T, Hoffmeister M, Brenner H. The underestimated impact of excess body weight on colorectal cancer risk: evidence from the UK Biobank cohort. Br J Cancer. 2023;129:829–37. 10.1038/s41416-023-02351-6 PMC10449928 37443347

[20] Zhan ZQ, Chen YZ, Huang ZM, Luo YH, Zeng JJ, Wang Y, et al. Metabolic syndrome, its components, and gastrointestinal cancer risk: a meta-analysis of 31 prospective cohorts and Mendelian randomization study. J Gastroenterol Hepatol. 2024;39:630–41. 10.1111/jgh.16477 38230882

[21] Rothwell JA, Jenab M, Karimi M, Truong T, Mahamat-Saleh Y, Ferrari P, et al. Metabolic syndrome and risk of gastrointestinal cancers: an investigation using large-scale molecular data. Clin Gastroenterol Hepatol. 2022;20:e1338–e1352. 10.1016/j.cgh.2021.10.016 PMC9117007 34687971

[22] Wang L, Lo CH, He X, Hang D, Wang M, Wu K, et al. Risk factor profiles differ for cancers of different regions of the colorectum. Gastroenterology. 2020;159:241–56.e13. 10.1053/j.gastro.2020.03.054 PMC7387153 32247020

[23] Siegel RL, Wagle NS, Cercek A, Smith RA, Jemal A. Colorectal cancer statistics, 2023. CA Cancer J Clin. 2023;73:233–54. 10.3322/caac.21772 36856579

[24] Spaander MCW, Zauber AG, Syngal S, Blaser MJ, Sung JJ, You YN, et al. Young-onset colorectal cancer. Nat Rev Dis Primers. 2023;9:21. 10.1038/s41572-023-00432-7 PMC10589420 37105987

[25] Kasprzak A. Insulin-like growth factor 1 (IGF-1) signaling in glucose metabolism in colorectal cancer. Int J Mol Sci. 2021;22:12. 10.3390/ijms22126434 PMC8234711 34208601

[26] Choi YJ, Lee DH, Han KD, Shin CM, Kim N. Abdominal obesity, glucose intolerance and decreased high-density lipoprotein cholesterol as components of the metabolic syndrome are associated with the development of colorectal cancer. Eur J Epidemiol. 2018;33:1077–85. 10.1007/s10654-018-0440-6 30196334

[27] Sarri G, Davies M, Lumsden MA. Diagnosis and management of menopause: summary of NICE guidance. BMJ. 2015;351:h5746. 10.1136/bmj.h5746 26563259

[28] Song CH, Kim N, Lee SM, Nam RH, Choi SI, Kang SR, et al. Effects of 17β-estradiol on colorectal cancer development after azoxymethane/dextran sulfate sodium treatment of ovariectomized mice. Biochem Pharmacol. 2019;164:139–51. 10.1016/j.bcp.2019.04.011 30981879

[29] Söderlund S, Granath F, Broström O, Karlén P, Löfberg R, Ekbom A, et al. Inflammatory bowel disease confers a lower risk of colorectal cancer to females than to males. Gastroenterology. 2010;138:1697–703. 10.1053/j.gastro.2010.02.007 20167217

[30] Tian Y, Kim AE, Bien SA, Lin Y, Qu C, Harrison TA, et al. Genome-wide interaction analysis of genetic variants with menopausal hormone therapy for colorectal cancer risk. J Natl Cancer Inst. 2022;114:1135–48. 10.1093/jnci/djac094 PMC9360460 35512400

[31] Baek C, Kim JE, Shin A, Choi JY. Association of menopausal hormone therapy with gastric and colorectal cancer risks in Korean women: A nationwide population-based cohort study. Maturitas. 2022;166:35–40. 10.1016/j.maturitas.2022.08.006 36055009

[32] Lin KJ, Cheung WY, Lai JY, Giovannucci EL. The effect of estrogen vs. combined estrogen-progestogen therapy on the risk of colorectal cancer. Int J Cancer. 2012;130:419–30. 10.1002/ijc.26026 21365647

[33] Veilleux A, Tchernof A. Sex differences in body fat distribution. In: Symonds ME, editor. Adipose tissue biology. Springer New York; 2012, p. 123–66.

