# Supplementary material for: Age- and sex-dependent associations of metabolic syndrome with colorectal cancer in the UK Biobank
Source: Br J Cancer. 2026 Jul 30;135(9):1463–9. doi: 10.1038/s41416-026-03564-1 (PMC13675222; doi:10.1038/s41416-026-03564-1)
Supplement: Supplementary file 1 [file 41416_2026_3564_MOESM1_ESM.docx]

**Supplementary Tables**

**Table S1. Definitions of metabolic syndrome components in the study.**

| **Metabolic syndrome components** | **Definition** |
| --- | --- |
| **Elevated waist circumference** | ≥ 102 cm in males, ≥ 88 cm in females |
| **Hypertriglyceridemia** | ≥150 mg/dL (1.7 mmol/L) |
| **Hyperglycemia** | Glycated hemoglobin (HbA1c) ≥42 mmol/mol, or antidiabetic medication use |
| **Elevated blood pressure** | Systolic ≥130 mmHg, diastolic ≥85 mmHg, or antihypertensive medication use |
| **Low high-density lipoprotein cholesterol (HDL-C)** | < 40 mg/dL (1.03 mmol/L) in males, < 50mg/dL (1.29 mmol/L) in females, or lipid-modifying medications |

**Table S2. Baseline characteristics and colorectal cancer event rates stratified by sex and age.**

|  | **Male, age<60**  (n=101,415) | **Male, age≥60**  (n=78,677) | **Female, age<60**  (n=117,078) | **Female, age≥60**  (n=82,003) | **P-value^a^** |
| --- | --- | --- | --- | --- | --- |
| Age, y | 50.6± (5.6) | 64.1± (2.8) | 50.7± (5.6) | 63.9± (2.8) | <0.001 |
| Ethnicity |  |  |  |  | <0.001 |
| White | 93,850 (93.0%) | 76,020 (97.2%) | 108,651 (93.2%) | 79,393 (97.2%) |  |
| Other | 7,043 (7.0%) | 2,217 (2.8%) | 7,912 (6.8%) | 2,308 (2.8%) |  |
| Townsend deprivation index | -1.0± (3.2) | -1.6± (3.0) | -1.2± (3.1) | -1.6± (2.9) | <0.001 |
| Educational qualifications |  |  |  |  | <0.001 |
| Higher academic/professional | 53,403 (53.2%) | 33,880 (43.7%) | 64,449 (55.6%) | 32,983 (40.8%) |  |
| Lower academic/vocational | 36,447 (36.3%) | 23,403 (30.2%) | 41,019 (35.4%) | 25,001 (30.9%) |  |
| None | 10,506 (10.5%) | 20,247 (26.1%) | 10,521 (9.1%) | 22,831 (28.3%) |  |
| Smoking status |  |  |  |  | <0.001 |
| Never | 55,300 (54.8%) | 32,596 (41.7%) | 72,021 (61.8%) | 47,104 (57.8%) |  |
| Former | 30,898 (30.6%) | 37,724 (48.2%) | 32,546 (27.9%) | 28,993 (35.6%) |  |
| Current | 14,797 (14.7%) | 7,875 (10.1%) | 12,015 (10.3%) | 5,440 (6.7%) |  |
| Alcohol consumption |  |  |  |  | <0.001 |
| Never | 22,406 (22.1%) | 23,107 (29.4%) | 17,289 (14.8%) | 14,621 (17.9%) |  |
| Special occasions only | 27,017 (26.7%) | 20,218 (25.7%) | 25,470 (21.8%) | 15,527 (19.0%) |  |
| 1-3 times a month | 28,015 (27.7%) | 18,651 (23.8%) | 31,676 (27.1%) | 19,595 (23.9%) |  |
| 1-2 times a week | 9,979 (9.9%) | 6,021 (7.7%) | 16,234 (13.9%) | 9,856 (12.0%) |  |
| 3-4 times a week | 7,230 (7.1%) | 5,793 (7.4%) | 16,283 (13.9%) | 13,377 (16.3%) |  |
| Daily or almost daily | 6,509 (6.4%) | 4,739 (6.0%) | 9,856 (8.4%) | 8,883 (10.9%) |  |
| Physical activity |  |  |  |  | <0.001 |
| Low | 16,791 (20.1%) | 10,933 (17.4%) | 17,725 (19.6%) | 8,889 (15.6%) |  |
| Moderate | 31,037 (37.2%) | 25,140 (39.9%) | 38,289 (42.4%) | 24,739 (43.3%) |  |
| High | 35,690 (42.7%) | 26,937 (42.8%) | 34,340 (38.0%) | 23,501 (41.1%) |  |
| Fruit intake (pieces/day) | 2.6± (2.5) | 2.9± (2.7) | 3.1± (2.5) | 3.6± (2.7) | <0.001 |
| Vegetable intake (tablespoons/day) | 4.5± (3.5) | 4.9± (3.4) | 5.0± (3.3) | 5.1± (3.1) | <0.001 |
| Red meat intake |  |  |  |  | <0.001 |
| Never | 5,471 (5.5%) | 2,732 (3.5%) | 11,451 (9.9%) | 5,420 (6.7%) |  |
| Less than once a week | 36,084 (36.0%) | 31,412 (40.4%) | 32,996 (28.5%) | 27,344 (33.7%) |  |
| Once a week | 22,025 (22.0%) | 16,843 (21.6%) | 25,047 (21.6%) | 17,698 (21.8%) |  |
| ≥2 times a week | 36,566 (36.5%) | 26,816 (34.5%) | 46,443 (40.1%) | 30,615 (37.8%) |  |
| Processed meat intake |  |  |  |  | <0.001 |
| Never | 5,961 (5.9%) | 3,662 (4.7%) | 15,276 (13.1%) | 9,607 (11.7%) |  |
| Less than once a week | 20,338 (20.1%) | 17,927 (22.9%) | 43,538 (37.3%) | 31,992 (39.1%) |  |
| Once a week | 29,131 (28.8%) | 24,300 (31.0%) | 33,183 (28.4%) | 23,694 (29.0%) |  |
| ≥2 times a week | 45,564 (45.1%) | 32,543 (41.5%) | 24,643 (21.1%) | 16,473 (20.1%) |  |
| CRC screening history | 16,050 (16.3%) | 39,746 (51.6%) | 17,891 (15.5%) | 40,048 (49.5%) | <0.001 |
| Family history of CRC | 10,014 (9.9%) | 9,892 (12.6%) | 10,897 (9.3%) | 10,101 (12.3%) | <0.001 |
| Use of NSAIDs | 27,127 (26.7%) | 30,954 (39.3%) | 33,895 (29.0%) | 25,645 (31.3%) | <0.001 |
| Event indicators |  |  |  |  |  |
| CRC cases | 962 | 1,907 | 815 | 1,287 | - |
| Incidence rate, per 100,000 person-years | 80.5 | 208.2 | 59.1 | 134.1 | - |
| Median follow-up time, y | 11.9 | 11.8 | 11.8 | 11.8 | - |
| Total follow-up time, person-years | 1,195,007 | 915,953 | 1,379,289 | 959,679 | - |

Data are presented as mean±(SD) or No. (%), or unless otherwise indicated. Abbreviations: MetS = metabolic syndrome; CRC = colorectal cancer; NSAIDs = nonsteroidal anti-inflammatory drugs.

^a^Kruskal-Wallis rank sum test; Pearson's Chi-squared test.

**Table S3. Interaction effects of metabolic syndrome with sex and age group on colorectal cancer risk.**

| **Interaction term** | **HR (95% CI)** | **P for interaction** | **Post-hoc power**^c^ |
| --- | --- | --- | --- |
| MetS × Sex^a^ | 1.06 (0.95, 1.20) | 0.303 | 0.169 |
| MetS × Age group^a^ | 0.93 (0.82, 1.05) | 0.217 | 0.236 |
| MetS × Sex × Age group^b^ | 1.02 (0.84, 1.22) | 0.860 | 0.042 |

Abbreviations: HR = hazard ratio; CI = confidence interval; MetS = metabolic syndrome.

^a^Adjusted for sex, age group (<60 vs ≥60), ethnicity, socio-economic deprivation, education, smoking status, alcohol consumption, physical activity, fruit, vegetable, red meat and processed meat intake, history of bowel cancer screening, family history of CRC, and regular use of NSAIDs.

^b^Adjusted for the combination of sex and age group, and all other covariates (excluding sex and age group).

^c^Post-hoc power was estimated for each interaction term using the observed HR, sample size, and α = 0.05. A threshold of 0.80 was used to indicate adequate power.
