# Supplementary material for: Age- and sex-dependent associations of metabolic syndrome with colorectal cancer in the UK Biobank
Source: Br J Cancer. 2026 Jul 30;135(9):1463–9. doi: 10.1038/s41416-026-03564-1 (PMC13675222; doi:10.1038/s41416-026-03564-1)
Supplement: Supplementary file 2 [file 41416_2026_3564_MOESM2_ESM.docx]

**Supplementary Figures**

**Figure S1. Sex-stratified age trends in the prevalence of metabolic syndrome.** Abbreviations: MetS = metabolic syndrome.

**Figure S2. Sex- and age-stratified variations of the prevalence of metabolic syndrome in the number of metabolic abnormalities.**

**Figure S3. Sex-stratified age trends in the prevalence of metabolic syndrome components.** Abbreviations: HDL-C = high-density lipoprotein cholesterol.

**Figure S4. Overall and sex- and age-specific hazard ratios and population attributable fractions for metabolic syndrome in colorectal cancer risk by tumour subsite.** Models were adjusted for age, sex (only in the analysis of total population), ethnicity, socio-economic deprivation, education, smoking status, alcohol consumption, physical activity, fruit, vegetable, red meat and processed meat intake, history of bowel cancer screening, family history of CRC, and regular use of NSAIDs. Abbreviations: CRC = colorectal cancer, HR = hazard ratio, CI = confidence interval, PAF = population attributable fractions.

**Figure S5. Overall and sex- and age-specific hazard ratios and population attributable fractions for metabolic syndrome in colorectal cancer risk by family history of colorectal cancer in first-degree relatives.** Models were adjusted for age, sex (only in the analysis of total population), ethnicity, socio-economic deprivation, education, smoking status, alcohol consumption, physical activity, fruit, vegetable, red meat and processed meat intake, history of bowel cancer screening, and regular use of NSAIDs. Abbreviations: CRC = colorectal cancer, HR = hazard ratio, CI = confidence interval, PAF = population attributable fractions.

**Figure S6. Overall and sex- and age-specific hazard ratios and population attributable fractions for metabolic syndrome in colorectal cancer risk across follow-up durations.** Models were adjusted for age, sex (only in the analysis of total population), ethnicity, socio-economic deprivation, education, smoking status, alcohol consumption, physical activity, fruit, vegetable, red meat and processed meat intake, history of bowel cancer screening, family history of CRC, and regular use of NSAIDs. Abbreviations: CRC = colorectal cancer, HR = hazard ratio, CI = confidence interval, PAF = population attributable fractions.

**Figure S7. Nonlinear association between metabolic syndrome biomarkers and colorectal cancer risk in males aged <60 years.** Nonlinear dose-response relationship analyzed using restricted cubic splines with knots at distribution quantiles (0.01, 0.50, 0.99). Models were adjusted for age, ethnicity, socio-economic deprivation, education, smoking status, alcohol consumption, physical activity, fruit, vegetable, red meat and processed meat intake, history of bowel cancer screening, family history of CRC, and regular use of NSAIDs. Abbreviations: HR = hazard ratio; HDL-C = high-density lipoprotein cholesterol.

**Figure S8. Nonlinear association between metabolic syndrome biomarkers and colorectal cancer risk in males aged ≥60 years.** Nonlinear dose-response relationship analyzed using restricted cubic splines with knots at distribution quantiles (0.01, 0.50, 0.99). Models were adjusted for age, ethnicity, socio-economic deprivation, education, smoking status, alcohol consumption, physical activity, fruit, vegetable, red meat and processed meat intake, history of bowel cancer screening, family history of CRC, and regular use of NSAIDs. Abbreviations: HR = hazard ratio; HDL-C = high-density lipoprotein cholesterol.

**Figure S9. Nonlinear association between metabolic syndrome biomarkers and colorectal cancer risk in females aged <60 years.** Nonlinear dose-response relationship analyzed using restricted cubic splines with knots at distribution quantiles (0.01, 0.50, 0.99). Models were adjusted for age, ethnicity, socio-economic deprivation, education, smoking status, alcohol consumption, physical activity, fruit, vegetable, red meat and processed meat intake, history of bowel cancer screening, family history of CRC, and regular use of NSAIDs. Abbreviations: HR = hazard ratio; HDL-C = high-density lipoprotein cholesterol.

**Figure S10. Nonlinear association between metabolic syndrome biomarkers and colorectal cancer risk in females aged ≥60 years.** Nonlinear dose-response relationship analyzed using restricted cubic splines with knots at distribution quantiles (0.01, 0.50, 0.99). Models were adjusted for age, ethnicity, socio-economic deprivation, education, smoking status, alcohol consumption, physical activity, fruit, vegetable, red meat and processed meat intake, history of bowel cancer screening, family history of CRC, and regular use of NSAIDs. Abbreviations: HR = hazard ratio; HDL-C = high-density lipoprotein cholesterol.
